# Understanding the value and impacts of informal care for people living with poor mental health

**DOI:** 10.1192/j.eurpsy.2022.289

**Published:** 2022-09-01

**Authors:** J. Saunders

**Affiliations:** EUFAMI, Family, Leuven, Belgium

**Keywords:** Recovery, caring, value

## Abstract

**Introduction:**

Our survey of more than 700 caregivers across Europe and Canada highlights the tremendous and too often hidden value of caregiving. In short informal carers are fundamental to the functioning of any health and social care system; it is critical to therefore to invest in measures to support these caregivers and identify potential risk factors that might lead to a breakdown in caregiving support.

**Objectives:**

To identify the importance of family care in the context of modern community mental health services.

**Methods:**

Survey questionaire and interview of family members. A survey was developed in consultation with EUFAMI.

**Results:**

The average length of the caring week exceeds the length of the working week On average informal carers provide more than 43 hours of care every week, well in excess of the average working week.

**Conclusions:**

Family care needs to be recognised as a significant part of the overall care package in differenct countries. Govenments need to acknowledge the real cost of care. In our report we have highlighted that the average caring week is much longer than the working week, and that this is over 60 hours per week for carers who live with the person that the care for. We have highlighted major detrimental effects on carer quality of life, as well as high levels of loneliness. We have also noted that more than a quarter of all carers have a depression or anxiety disorder. We have seen wider adverse impacts on potential career and education prospects as well as financial worries.

**Disclosure:**

This survey and report were possible thanks to the sponsorship of Ferrer Internacional S.A, Janssen Pharmaceutica NV, Lundbeck A/S and Otsuka Pharmaceutical Europe Ltd. The sponsors did not have any influence over the content

